# Position-context additive transformer-based model for classifying text data on social media

**DOI:** 10.1038/s41598-025-90738-1

**Published:** 2025-03-08

**Authors:** M. M. Abd-Elaziz, Nora El-Rashidy, Ahmed Abou Elfetouh, Hazem M. El-Bakry

**Affiliations:** 1 Information Systems Department, Faculty of Computers and Information Sciences, Mansoura University, Mansoura, Egypt; 2 Machine Learning and Information Retrieval Department, Faculty of Artificial Intelligence, Kaferelshikh University, Kafr El Sheikh, Egypt; 3 Delta Higher Institute for Management and Accounting Information Systems, Mansoura, 35511 Egypt

**Keywords:** Social media, Transformer-based model, Word embedding, Bi-LSTM network, Additive attention, Computer science, Information technology

## Abstract

In recent years, the continuous increase in the growth of text data on social media has been a major reason to rely on the pre-training method to develop new text classification models specially transformer-based models that have proven worthwhile in most natural language processing tasks. This paper introduces a new Position-Context Additive transformer-based model (PCA model) that consists of two-phases to increase the accuracy of text classification tasks on social media. Phase I aims to develop a new way to extract text characteristics by paying attention to the position and context of each word in the input layer. This is done by integrating the improved word embedding method (the position) with the developed Bi-LSTM network to increase the focus on the connection of each word with the other words around it (the context). As for phase II, it focuses on the development of a transformer-based model based primarily on improving the additive attention mechanism. The PCA model has been tested for the implementation of the classification of health-related social media texts in 6 data sets. Results showed that performance accuracy was improved by an increase in F1-Score between 0.2 and 10.2% in five datasets compared to the best published results. On the other hand, the performance of PCA model was compared with three transformer-based models that proved high accuracy in classifying texts, and experiments also showed that PCA model overcame the other models in 4 datasets to achieve an improvement in F1-score between 0.1 and 2.1%. The results also led us to conclude a direct correlation between the volume of training data and the accuracy of performance as the increase in the volume of training data positively affects F1-Score improvement.

## Introduction

In the past few years, deep learning has emerged as one of the most powerful and successful research areas in machine learning. It is commonly used to solve complex problems such as image recognition, text classification, etc. To effectively represent inputs into a neural network (e.g., words), it is important to have a good understanding of their context importance.

Word embedding is a technique for representing text using vectors. Vector representation of words allows for better understanding and analysis of the meaning of individual words and phrases. The most common vector representations techniques are word2vec and GloVe^1^, both trained on large amount of data. There has been recent development in the field of deep learning which has made it possible to train end-to-end models that can learn multiple layers (including features) rather than just one layer as used in traditional machine learning approaches such as supervised classifiers or unsupervised neural networks with generalization error being proportional to number features learned (i.e., lower dimensionality). Undoubtedly, the focus on developing word embedding technology has helped to improve the accuracy of machine learning algorithms^2,3^. The main advantage of using word embedding is that it can reduce the amount of data that needs to be processed. For example, if you are trying to learn to recognize a dog breed, you might not need to learn the individual dog breeds. Instead, you could use word embedding to represent each breed as a vector in a space. This would allow the machine learning algorithm to learn to recognize the dog breed without having to learn the individual dog breeds.

Word position is one of the most important features in word embedding for deep learning. Word positions play a critical role in representing meaning of words and making them accessible for machine learning models. Various studies have shown that when different types of data are processed with more accurate representations, it leads to better performance by the machine-learning algorithms^4,5^. In more details, the positional information refers to the specific location of each word in a text or sentence. By considering the position of words, it is possible to improve the accuracy of a machine learning model by better recognizing the relationships between words. There are several ways to adjust word embedding to improve deep learning performance^6–10^. This includes adjusting the vector orientation, the number of dimensions, and the co-occurrence matrix. On the other hand, context can play an important role in providing better representation of inputs, reflected in the increased accuracy of deep learning models’ performance.

Based on these challenges, this study aims to address the following research questions (**RQs**):

- **RQ1:** How to improve the process of word representation based on the contextual and positional features of the input text and determine how it affects the upgrading of the classification process?
- **RQ2:** How to develop attention layer performance and study the extent of its impact on the performance of the proposed transformer-based model compared to the pre-trained models that have achieved the best results in recent years?

The main contribution of this research is to answer the research questions RQ1- RQ2 by developing the Position-Context Additive (PCA) transformer-based model to improve the accuracy of the text classification on social media. The proposed PCA model employs a two-phase approach:

- **Phase 1:** Focuses on enhancing word embedding techniques to improve the representation of text data. This is achieved by concatenating two vectors:
  - **Positional Vector:** Each word’s position in the input sentence is represented by a unique positional vector.
  - **Contextual Vector:** This vector created using a Bi-LSTM (Bidirectional Long Short-Term Memory) network that processes the relationships between words in both forward and backward passes. This combination provides richer representation by capturing the word’s semantic similarity and its position in the context, enabling the model to focus on the relevance of the surrounding words.
- **Phase 2:** Enhances the additive attention mechanism in the transformer model to improve classification efficiency. This is done by:
  - **Improved Global Attention:** Rather than relying on the traditional dot-product attention mechanism, the model introduces a global query and context-aware key mechanism to summarize the attention query and key matrices. This helps in modeling long sequences more efficiently by capturing more complex relationships between tokens.
  - **Multi-head Attention:** Using 16 attention heads allows the model to learn diverse aspects of the text representation simultaneously.

Finally, the significant contributions of this research and potential answers to research questions (ARQs) are as follows:

- **ARQ1**: Improving the word embedding process by using two vectors to express each word in the input layer, the first vector represents the positional information that concatenated to the initial Word2Vec representation of inputs and then uses two layers of the Bi-LSTM network to reflect the contextual information.
- **ARQ2**: Improving the accuracy of the proposed model’s performance compared to pre-trained transformer-based models^11–13^. It was implemented by improving the attention layer of the proposed additive transformer model through modelling the global contexts then transform the representation of tokens depending on the interaction between their global representation instead of pair-wise tokens interaction.

This paper is organized as follows. Section “Related work” presents related work regarding classification and transformer-based models. Section “Materials and methods” describes the materials and methods used to analyse text classification tasks. Section “Results” presents our results, and Section “Discussion” discusses them. Finally, Section “Conclusion” offers our conclusions.

## Related work

Recently, the focus has increased on developing deep learning-based models to deal with natural language processing (NLP) tasks. A new model based on convolutional neural network was proposed^14^, this model was based on the use of a context-dependent and lexicon-based to detect hidden features and the sentiment analysis strength. The empirical results demonstrated the high efficiency of the proposed model to predict the strength of sentiment compared to the baseline methods. Finally, the proposed model performed higher quality than current lexicons. In^15^, new hybrid model was proposed to extract the text features, this model presented the integration between the representations of bi-directional encoder in the transformer model and the propagation of news as a graph neural network. The results of that hybrid model showed more accuracy in extracting context features with F1-score = 0.91 on PolitiFact dataset and F1-score =0.93 on the Gossipcop dataset, respectively.

A comparative study in^16^ was presented to detect fake news about COVID-19 using models that based on transformer such as BERT, BERT without LSTM, ALBERT, RoBERTa, this paper also proposed a hybrid model that merge BERT & ALBERT models for detecting both real and fake news, All models had shown good results while RoBERTa model had achieved more accurate results (F1-score of 0.98) in both real and fake classes. likewise, the performance of pre-trained transformer models has been measured in^17^ using 25 datasets, 6 of which are health related. RoBERTabase, BERTweet and ClinicalBioBERT language models were compared based on classification accuracy. Experiments showed that RoBERTa-base and BERTweet performed relatively well in most datasets, much better than ClinicalBioBERT, even in health-related datasets.

BiLSTM model with a multipolar attention mechanism had been proposed in^18^ to analyse implicit emotions where a perpendicular limitation technique is adopted to guarantee that the distinctive performance can be maintained during optimization. The proposed model was trained in 2 datasets, one explicit dataset and the other was implicit sentiment analysis dataset SMP2019. The results showed that the model captured the discriminatory differences between sentiment polarizations accurately. In^19^, a new attention-based model focused on improving the calculation of ideal attention weights in the training phase had been proposed through future information rather than using the hidden state of current inputs, then using these weights to solve the problem of attention deviation. This model had been experimented on MSCOCO and Flicker30k datasets and the results showed that the proposed model exceeded baseline models based on two criteria human assessments and automated metrics. Table 1 reviews a summary of the related work in terms of the data sets used, their sizes, the methodology applied, how they are evaluated and results.

**Table 1:** The summary of the related work.

References	Methodology	Datasets	Evaluation	Results
Minghui et al.^14^	Lexicon-based CNN	6 datasets: LSD: 4509—Opinion Lexicon: 7000 IMDB: 50,000—Sentiment lexicon: 6800—AFINN: 3300—first GOP debate: 14,000	Compared to 7 lexicons models competitions	It performed higher quality than current lexicons models
SaiKia et al.^15^	A hybrid graph neural network-based approach	Politifact dataset: News Articles: 1056 Tweets: 564,129—Unique Users: 798,183 Gossipcop dataset: News Articles: 22,140 Tweets: 1,396,548—Unique Users: 703,669	Compared to 4 baseline models	F1-score = 0.91 on PolitiFact dataset and F1-score = 0.93 on the Gossipcop dataset
Sajib et al.^16^	Comparative study	COVID-19 dataset: 10,700 social media items and articles	Compared to 5 models: BERT, BERT without LSTM, ALBERT, RoBERTa, a Hybrid of BERT & ALBERT	The RoBERTa model achieved more accurate results, F1 score of 0.98
Yuting et al.^17^	Comparative study	25 social media text classification datasets: OLID-1: 12,776—OLID-2: 12,156—OLID-3: 12,129 TRAC-1-1:12,915—TRAC-1-2:13,256- TRAC-2-1: 5463 TRAC-2-2:5463—Sarcasm-1: 5760—Sarcasm-2: 6300 CrowdFlower: 36,808 -FB-arousal-1: 2665 FB-arousal-2: 2678—FB-valence-1: 2659 FB-valence-2: 2670—SemEval-18-A: 2703 SemEval-18-F: 3238—SemEval-18-J: 2721 SemEval-18-S: 2508—SemEval-18-V: 2120 ADR Detection: 5470—BreastCancer: 4717 PM Abuse: 15,100—SMM4H-17-task1: 11,605 SMM4H-17-task2: 13,220—WNUT-20-task2: 7238	Benchmark the performances of 3 models, RoBERTa-base, BERTweet and ClinicalBioBERT	RoBERTa-base and BERTweet perform comparably on most datasets, and considerably better than ClinicalBioBER model
Jiyao et al.^18^	BiLSTM model with multi-polarity orthogonal attention for implicit sentiment analysis	2 Datasets: SMP2019: 13,525 EWECT: 14,638	Compared to 4 baseline models	The proposed model was more accurately captured the characteristic differences among sentiment polarities
Fenglin et al.^19^	Prophet Attention: Predicting Attention with Future Attention	2 Datasets: MSCOCO: 123,287 Flickr30k: 31,783	Compared to 3 representative models Up-Down, GVD and AoANet model	It achieved the 1st place on the leaderboard of the online MSCOCO benchmark in terms of the default ranking score

## Materials and methods

### Methodology

This paper introduces a new Position-Context Additive transformer-based model (PCA model) that consists of two-phases to enhance the model accuracy. This is done by providing the best representation of the model inputs by focusing on the importance of the words position in the input sentence as well as the importance of the meaning of the words within the context. On the other hand, the model adds improvements to the attention step in the transformer model to help increase the model accuracy and the speed of learning at the training step.

**Fig. 1:**
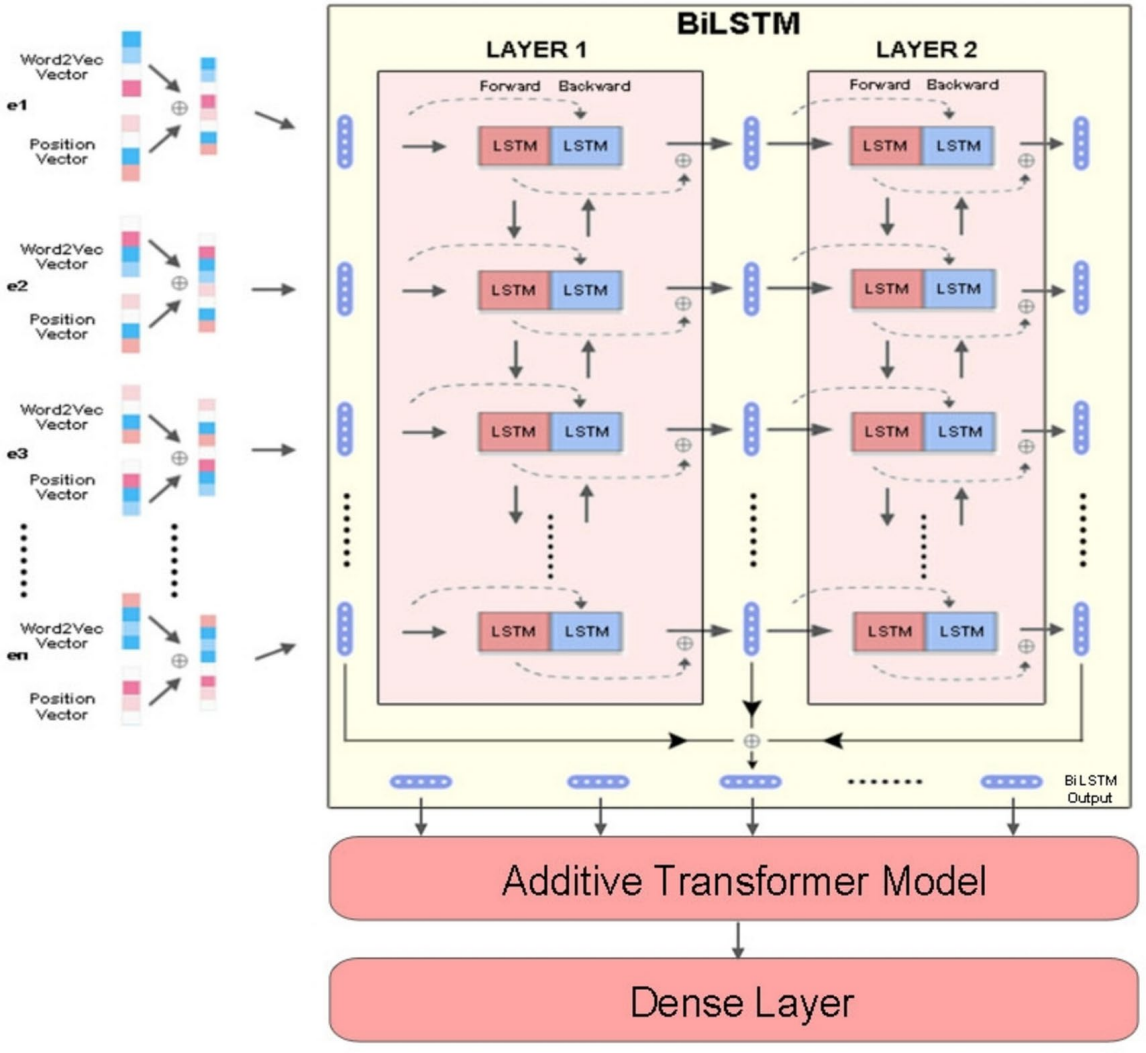
Proposed methodology for the PCA model.

As mentioned before, the proposed model consists of two phases. The first phase aims to achieve **ARQ1** by developing a new methodology for extracting text features by paying attention to position and context for each word in the input layer. The essence of attention to a word position is to improve the word embedding step by using two vectors to express each word in the input layer. The Word2Vec method was used to represent the initial first vector while the relative position representation method was used for the second vector, then the two vectors are concatenated into one vector that would be the input to the Bidirectional Long Short-Term Memory (BiLSTM) network. Figure 1 shows the PCA model methodology.

The proposed model begins with the word embedding process through representing each word of the input sentence with 300-dimensional initial vector by relying on the Word2Vec method to capture semantic similarities between words, such that words appearing in similar contexts tend to have similar vector representations. In addition, a table of 128 vectors is also created, which is the maximum size of the allowable input sentence where a random 300-dimensional vector is used to represent the position of a word in the sentence such as position 1, position 2, …, position128. Following this, the initial vector (Word2Vec vector) is concatenating (end-to-end) with the positional vector from the created positions table according to the word order in the input sentence to produce a new 600-dimension vector that achieves a richer, more nuanced representation of each word, encapsulating not only its semantic similarity to other words but also its unique position within a specific textual context, so even if the same word is repeated in the entry sentence, its representation will be different as a result of the different position vector.

Relying on the Word2Vec method may give rise to the problem of out of vocabulary (OOV), meaning that the word does not exist in the embedding model. The proposed model deals with this problem by using sub-word tokenization (character level) to represent OOV words by breaking them into smaller units in the vocabulary to extract their features and then summed up together to form OOV word features^20^.

On the other hand, attention was paid to context using BiLSTM network that composed of two layers where each layer contains two passes, one forward and the other backward. the first layer receives concatenated vectors as an input, the forward pass includes information about particular word and the context for the other words before that word. And in the opposite way, the backward pass includes information about the word and the context after it. This information is used to construct the first intermediate word vectors which is used as input to the next layer. In the same way, the same task is repeated in both directions in the second layer to produce the second intermediate vectors. Finally, the output vectors are the weighted sum of the input vectors and the two intermediate vectors.

The second phase depends on the development of a transformer-based model to achieve **ARQ2** by developing the additive attention mechanism that has proven highly efficient to understand texts especially in long sequences. The model starts by receiving the vector matrices we got from the first phase and transform them into the query, key and value sequences that represent input vector elements E= [e_1_, e_2_, …, e_N_] and symbolizing them with E ∈ R ^N × d^, where N is the length of the sequence and d is the hidden dimension. In this model we will use 16 attention heads, in each head we will use 3 transformation layers, one to query where Q = [q_1_, q_2_, …, q_N_], the second to key where K = [k_1_, k_2_, …, k_N_], and the third layer for value where V = [v_1_, v_2_, …, v_N_]. Now we focus our attention on solving the problem of inefficiency in modelling the long sequence by replacing the dot-product mechanism with another mechanism in the process of modelling the information fed from the first phase through the interactions between attention query, key, and value. This is done by summarizing the query matrix in a vector representing the general query. This step begins with calculating the weight of attention α_*i*_ for *i*-th query vector through Eq. 1 as follows:

1 $${\alpha }_{i}=\frac{\text{exp}( {W}_{q}^{T}{q}_{i}/ \sqrt{d})}{{\sum }_{j=1}^{N}\text{exp}( {W}_{q}^{T}{q}_{j}/ \sqrt{d})}$$

where *W*_*q*_ is the vector of the learnable parameter that generated by weight generator technique in Eq. 2:

2 $$Vd{W}_{q(i+1)}= \rho Vd{W}_{q(i-1)}+\left(1-\rho \right)d{W}_{qi}$$

This equation is used to calculate the exponentially weighted averages and the change in deriving weights in every iteration based on a percentage of the previous deriving weight VdW_*q*(*i−*1)_ and is represented by the ρ coefficient and a percentage of the current deriving weight VdW_*q i*_ where ρ = 80%. Then, by using the weight of attention α_*i*_, the vector that represents the attention of the global query can be calculated through Eq. 3 as follow:

3 $$q= \sum_{i=1}^{N}{\alpha }_{i }{q}_{i}$$

On the other hand, the interactions between the vector representing the global query and each vector representing the key are modelled into a global context-aware key matrix through the Eq. 4 as follows:

4 $${p}_{i}=q* {k}_{i}$$

where p_*i*_ is the global key matrix, * is the element-wise product. Using the same mechanism, we summarize the global key matrix and use the additive attention to calculate each vector’s weight through the Eq. 5 as follows:

5 $${\beta }_{i}=\frac{\text{exp}( {W}_{k}^{T}{p}_{i}/ \sqrt{d})}{{\sum }_{j=1}^{N}\text{exp}( {W}_{k}^{T}{p}_{j}/ \sqrt{d})}$$

where *W*_*k*_ is the learnable parameter that generated by the weight generation technique in equation 3 to represent key weights matrix, Eq. 6 is used to compute the global key vector k:

6 $$k= \sum_{i=1}^{N}{\beta }_{i }{p}_{i}$$

The next step is to calculate *u*_*i*_ that represent the interaction between k (global key vector) and *v*_*i*_ (attention value matrix) using Eq. 7 as follows:

7 $${u}_{i}=k* {v}_{i}$$

Then we learn a hidden representation by using a linear transformation layer for each vector that represents the interaction between key and value. Finally, the final output is due to the addition of the output matrix R = [r_1_, r_2_, …, r_N_] to the query matrix. The last layer is the dense layer that is fed from the final output of the second phase, the function of this layer is to obtain a probability distribution over the classes by using sigmoid activation for binary classification and softmax for multiple classification.

### Data

We have used 6 health-related datasets to experiment our proposed model, where all datasets were published and authorized for use, that were contained tasks such as detecting of the adverse drug reaction (ADR). also, detect tweets that mentioned the medication to identify the medication consumption and Identify informative COVID-19 related tweets (WNUT), and 4 SMM4H tasks. The detailed data about datasets are described in Table 2.

**Table 2:** The detailed data about datasets.

Dataset	Training set	Testing set	No. of classes
ADR Detection	4318	1152	2
WNUT-20-task2	7842	2158	2
SMM4H-17-task1	7791	5382	2
SMM4H-17-task2	15,324	5000	3
SMM4H-18-task3	23,403	5000	2
SMM4H-18-task4	6956	1610	2

ADR detection is the adverse reaction of drug dataset that created by^21^ and published in^22^, it’s a binary classification prepared to detect the mention of adverse drug effects in twitter. WNUT-20-task2: it’s a dataset provided from Workshop on Noisy User-generated Text (W-NUT) in 2020 by^23^ where data published in^24,25^, it contains 10000 English tweets related to COVID-19, the purpose of the task2 is to detect tweets that contain informative information about COVID-19.

SMM4H-17 task1 and task2 are the Social Media Mining for Health Applications datasets in 2017 by^26^ that published in^27–29^, its prepared to analyse the mention of adverse drug effects in twitter. Task 1 is a binary classification to detect whether tweets mentioned drug name or dietary supplement or not as defined by the United States Food and Drug Administration (FDA). Task 2 is used to classify tweets that describe taking the drug. It contains 3 classes, one for the tweets that clearly express taking a personal drug, the second class is for tweets that may have mentioned taken the drug, and the third class is for the tweets that does not refer to the personal intake but mentioned the names of the drugs.

SMM4H-18 task3 and task4 are the Social Media Mining for Health Applications datasets in 2018 by^30^ that published in^31,32^. Task 3 is a binary classification that detect tweets whether it contain ADR or not . Finally, Task 4 is a binary classification to detect whether tweets mentioned that someone received, or intended to receive a flu vaccine, it deals with the automatic detection of posts mentioning vaccination or not.

### Data preprocessing

The data on Twitter contains a lot of noise. Therefore, we have taken pre-processing steps for all datasets to help pre-trained models achieve better performance^33,34^. we start with Unescape HTML tags, removed all non-alphanumeric characters, removed whitespaces such as tabs and newlines, hyperlinks that mentioned in the tweets replaced with URL, replaced emojis with a short textual description by using the Python emoji library. Finally, implemented lowercasing, normalizing numbers, capital words and repeated letters.

Initially, the Keras2.4 (Python deep learning API) was used to develop the PCA model with TensorFlow backend. The data were processed and prepared using the Scikit-learn library. As for training the model we used an 8-core CPU due to the small volume of training data. The hyperparameters of the PCA model are identified as follows, we trained the PCA model for 3 epochs with 3e-5 as a learning rate using Adam optimizer. The 10% of the training steps are used as warm-up steps and we set 64 as a batch size and 128 as a maximum sequence size. We used 0.5 as threshold for the classification tasks. For the BiLSTM network we set the hidden states to 300 (150 forward and 150 backward), The dropout rate is set to 0.3 on top of the LSTM layers. We trained the model to minimize the loss function using stochastic gradient descent in the back-propagation step. For the fine-tuning steps, we used softmax function as an output activation function and used the sigmoid function in the pretrained steps for the multilabel classification tasks.

## Results

We applied the PCA model on the test sets for 6 health related datasets, all tasks are evaluated using precision, recall and F1-score for the positive class except SMM4H-17 task 2 that use the micro-averaged F1-score for classes 1 and 2. The evaluation results was listed in Table 3 and compared to the best results that have been published in social media mining for health applications workshop that are achieved by the research teams participating in the classification tasks.

**Table 3:** The evaluation of PCA model results compared to the best results.

Dataset	Model	P	R	F1-score
ADR Detection	PCA model	0.927	0.909	0.918
	Best results	0.852	0.784	0.816
WNUT-20-task2	PCA model	0.952	0.876	0.912
	Best results	0.958	0.867	0.910
SMM4H-17-task1	PCA model	0.952	0.928	0.939
	Best results	0.937	0.891	0.914
SMM4H-17-task2	PCA model	0.651	0.798	0.717
	Best results	0.654	0.783	0.713
SMM4H-18-task3	PCA model	0.927	0.918	0.922
	Best results	0.931	0.864	0.896
SMM4H-18-task4	PCA model	0.814	0.890	0.850
	Best results	0.791	0.923	0.852

It was important to compare our model performance in terms of classification accuracy with pre-trained transformation-based models that have achieved promising results in recent years, including RoBERTa-base, BERTweet and ClinicalBioBERT model.

The RoBERTa-base model was proposed by^35^, and had the same architecture as the BERT model, but the performance of this model was more advanced as a result of adding several improvement steps to the BERT model. The most important of these steps are training the model on a larger volume of data, deleting the expected sentence during the pre-training process, and implementing the dynamic mask technique.

BERTweet’s model was developed by^36^, it based on the use of the RoBERTa structure for pre-training the model on a huge range of English tweets that feature the use of informal rules. It should be noted that the BERTweet model was also able to overcome other NLP models in extracting English tweet features where it achieved better results in 3 tasks such as text classification, named entity recognition label (NER) and part speech marking (POS).

The ClinicalBioBERT model was proposed by^37^ to develop the BioBERT model by using clinical observations for further training to extract clinical knowledge, and the results of the experiments also showed that the ClinicalBioBERT model was able to overcome the BERT base and other models in many medical-related NLP tasks such as entity recognition and natural language inference.

The results of comparing our model’s performance with the other three models in terms of F1-score are shown in Table 4.

**Table 4:** The F1-score comparison of the 4 models performance.

Dataset	RoBERTa-base	BERTweet	Clinical BioBERT	PCA model
ADR Detection	91.4	91.7	90.4	91.8
WNUT-20-task2	89.1	88.3	86.5	91.2
SMM4H-17-task1	93.6	93.5	92.7	93.9
SMM4H-17-task2	78.4	79.9	75.1	71.7
SMM4H-18-task3	89.7	91.7	90.8	92.2
SMM4H-18-task4	85.2	84.6	86.1	85.0

Ablation study was also presented in Table 5, based on the SMM4H-17-task1 dataset as an example, to illustrate the importance of each step of the proposed model and its impact on the model’s data classification performance.

**Table 5:** The ablation study for the proposed model.

Model	Precision	Recall	F1-Score
Work2Vec vector + Position vector + BiLSTM + Additive transformer	0.952	0.928	0.939
Work2Vec vector + BiLSTM + Additive transformer	0.923	0.900	0.911
Work2Vec vector + Position vector + Additive transformer	0.902	0.880	0.891
BiLSTM + Additive transformer	0.916	0.893	0.904

## Discussion

The results of the experiments showed that PCA transformer-based model achieved promising accurate results in classifying texts on social media, enabling it to overcome the models that achieved the best results published in SMM4H applications workshop. Furthermore, comparison of results in Table 3 showed that PCA model has overcome the other models in five out of six tasks: ADR Detection, WNUT-20-task2, SMM4H-17-task1, SMM4H-17-task2 and SMM4H-18-task3 respectively.

For the ADR detection dataset, our model achieved an increase in precision of 7.5% while the increase in recall was 12.5%, meaning that the F1-score has improved by about 10.2% from the best published results. Moreover, in WNUT-20-task2 dataset, the PCA model achieved a 0.9% higher recall rate but unfortunately with a 0.6% lower precision rate, reflecting a 0.2% higher F1-score rate than the best results. In the SMM4H-17-task1 dataset, the results of the experiment showed that PCA model beat the best results to achieve 93.9% in F1-score with an improvement of 2.5% after an improvement in precision of 1.5% and 3.7% increasing in the recall rate.

**Fig. 2:**
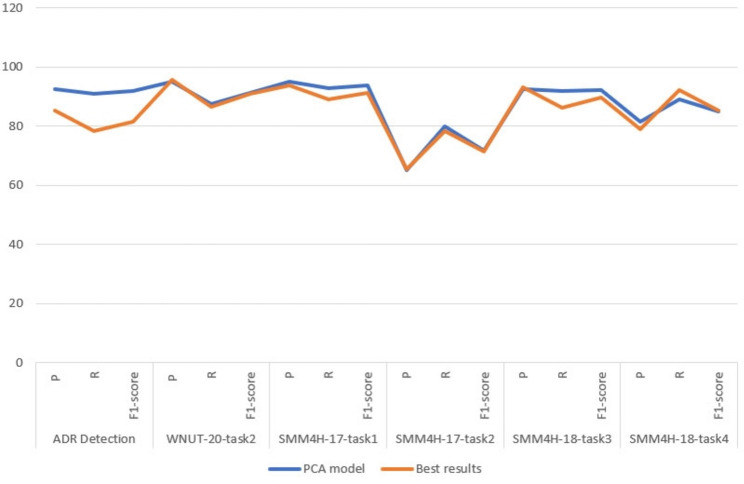
Illustrate the PCA model performance compared to the best results in 6 datasets.

In the same way, the PCA model had the ability to surpass the best results for Task 2 in the SMM4H-17 dataset, it exceeded the F1-score by 0.4% compared to the F1 result for the best results, mainly due to the remarkable improvement in the recall, which was 1.5%. Moreover, the results of the PCA model of Task 3 in a dataset SMM4H-18 achieve a recall rate of 91.8%, which exceeds the recall rate of best results by 5.4%, which significantly contributed to an increase in the F1-score rate by 2.6%, despite the lack of precision by 0.4%. Finally, the results of the PCA model in Task 4 of the SMM4H-18 dataset came close to exceeding the best results. The F1-score rate has achieved 0.2% less than the best results. This was due to a 3.3% drop in recall. Furthermore, compare the performance of the PCA model with the models that achieved the best results shown in Fig. 2.

**Fig. 3:**
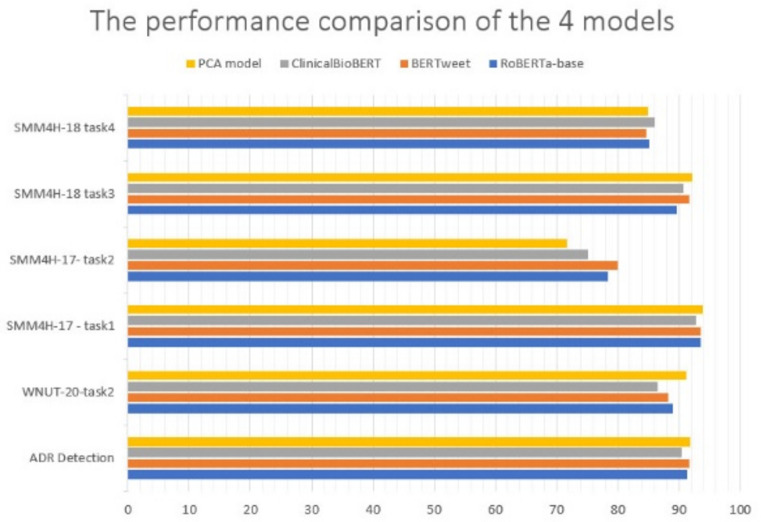
Illustrate the comparison between the performance of the three models and the PCA model.

On the other hand, the performance of the PCA model using a F1-score parameter has been compared with three other pre-trained transformer models on 6 social media datasets to perform the text classification tasks. Figure 3 shows the graph showing the comparison of PCA model performance with the best transformer-based models.

In other words, the comparison details are shown in Table 4, where the competition was strong among the four models, our model was able to overcome the others in four datasets: ADR Detection, WNUT-20-task2, SMM4H-17-task1 and SMM4H-18-task3, respectively.

The PCA model successfully classified the ADR Detection dataset by F1-score = 91.8%, surpassing other models and exceeding the best BERTweet model F1-score by 0.1%. It also achieved F1-score = 91.2% which exceeded the nearest competing model, the RoBERTa-base 2.1% in the WNUT-20-task2 dataset. With the same way, The PCA model beat the other three models in the SMM4H-17-task1dataset, where the PCA model achieved F1-score = 93.9% to exceed the best results of the RoBERTa-base model by 0.3%, and it achieved F1-score = 92.2% in SMM4H-18-task3 dataset to beat the other models and exceeding the best BERTweet model F1-score by 0.5%. Unfortunately, the PCA model has not been able to outperform the rest of the models in other datasets. It ranked third in the SMM4H-18-task4 datasets by 85%, as well as fourth in the SMM4H-17-task2 dataset by 71.7%.

The results of the ablation study in Table 5 show that the model based on Position-Context Additive transformer based model (PCA model) has been able to classify the SMM4H-17-task1 dataset with high efficiency, achieved a rate of 93.9% at the F1-score , exceeding the best results model that mentioned in Table 3 for the same dataset by 2.5%, but when dispensing with the position vector to represent the data, the F1-score decreased to 91.1%, so it is below the best model rate of 0.3%. On the other hand, when abandoning BiLSTM, we find that the F1-score rate has dropped to 89.1% to be 2.3% lower than the best results model, and ultimately, when relying on BiLSTM with additive transformer model the result of F1-score was 90.4% which is 1% lower than the F1-score of the best model.

### Error analysis

Previous results showed that the proposed PCA model has often been able to achieve high classification efficiency, compared to the best results models and the transformer-based models that have achieved the best results in recent times. But disappointingly, the proposed model could not overcome the best results model in accomplishing task4 of the SMM4H-18 dataset in Table 3 and the Clinical BioBERT model in Table 4, respectively. The proposed model results were 0.02% lower than the best results in Table 3, and 1.1% lower than the Clinical BioBERT model, which achieved the best results in Table 4. By analysing the causes of underperformance in this task, we found that the proposed model’s ability to achieve 2.3% higher precision than the best results model, as precision represents a criterion for measuring the efficiency of the classification models. In more detail, the actual positive results were 985 records, the proposed model was able to predict true positive results representing 802 records while the false positive results represented 183 records. This helped the proposed model to predict true positive results that outperform the predicted true positive results of the best results model at 2.3%.

On the other hand, the proposed model predicted false negative results by 99 records, affecting the recall rate to be 89%, which exceeds the best results model by 33 records, that achieved a recall rate of 92.3%, which contributed to reducing the F1-Score rate of the proposed model to be 85%, that is less than the best results model by 0.2%.

Similarly, the results of the PCA model in Task 2 of the SMM4H-17 dataset in Table 4 were unable to overcome the results of other pre-trained transformer-based models, as the proposed model achieved an 8.2% lower F1-score rate than the BERTweet model that represents the best results. By examining the reasons for this decline, we found that there were 3,867 records representing actual positive results, the proposed model was able to predict true positive results representing 2854 records while the false positive results represented 1013 records. This led to an increase in the precision of the proposed model compared to other models, but on the other hand, the proposed model’s ability to predict false negative results by 1241 records led to reduce the recall of the proposed model to 69.7%, which directly affecting on reducing the F1-Score rate of the proposed model to 71.7%, less than the BERTweet model.

Finally, based on previous results analyses, there are many reasons why the proposed model is unable to overcome other models in SMM4H-17-task2 and SMM4H-18-task4 datasets, such as texts in which the meaning is heavily dependent on the broader context, As an example the post: “Ah, my allergies are off the charts today!” and the follow up post: “Never mind, I found my relief in a tiny pill. The problem that the model without the context of the previous post may not realize that the “tiny pill” refers to allergy medication. Besides, there is no well representation of niche abbreviations and acronyms in training data, for example, the post: “My PCP recommended SSRIs for my anxiety. Feeling hopeful.”, misclassification may occur because the model could not identify ‘PCP’ (primary care physician) or ‘SSRIs’ (Selective Serotonin Reuptake Inhibitors). In addition, texts containing topics specialized in a field, such as medicine, are not adequately represented in training data, as an example the post: “After starting biotherapy for my RA, I noticed significant improvements. #rheumatoidarthritis”, the problem occurred due to “biologic therapy” and “RA” (Rheumatoid Arthritis) are technical terms that might not be well-represented in general training data.

## Conclusion

In this work, we have introduced an improved transformer-based model that based on two main axes: the first is about improving the text embedding process and the other focuses on increasing the accuracy of text classification. We have also applied this model to 6 text classification tasks to classify health-related texts on social media. Results showed that our improved model introduced the state-of-the-art performance in 5 of the 6 classification tasks compared to the models that achieved the best results, it provided better performance accuracy in five datasets: ADR Detection, WNUT-20-task2, SMM4H-17-task1, SMM4H-17-task2 and SMM4H-18-task3 datasets to be an improved accuracy ratio of 10.2, 0.2%, 2.5%, 0.4% and 2.6% respectively. The results also demonstrated by comparing the developed PCA model with three of the best pre-training transformer-based models: RoBERTa-base, BERTweet and ClinicalBioBERT models that PCA model has achieved positive results in 4 out of 6 of the text classification tasks as it outperformed the three models in the ADR Detection, WNUT-20-task2, SMM4H-17-task1 and SMM4H-18-task3 datasets at 0.1%, 2.1%, 0.3% and 0.5% respectively.

Analysing lack of performance of the proposed PCA model in the classification process for SMM4H-17-task2 and SMM4H-18-task4 datasets where the best results are not overcome. This may be due to several reasons, including that the text used in the training process can be misclassified if the model evaluates it in isolation from other texts that interpret it in which meaning depends heavily on the broader context, such as a series of messages or publications. Also, the text of the training can also contain medical abbreviations and uncommon expressions where model cannot easily identify them, thereby increasing the error rate in the classification process. Based on previous analyses, further training of the proposed model on more specialized medical terminology may contribute effectively to enhancing its efficiency in the classification of texts.

## Data Availability

The datasets used and/or analysed during the current study are available from the corresponding author on reasonable request.
